# Does self-modulated learning vs. algorithm-regulated learning of dermatology morphology affect learning efficiency of medical students?

**Published:** 2019-07-24

**Authors:** Danya Traboulsi, Jori Hardin, Laurie Parsons, Jason Waechter

**Affiliations:** 1 Division of Dermatology, Department of Medicine, University of Calgary, Alberta, Canada; 2 Departments of Critical Care and Anesthesiology, University of Calgary, Alberta, Canada

## Abstract

**Background:**

Deliberate practice is an important method of skill acquisition and is under-utilized in dermatology training. We delivered a dermatologic morphology training module with immediate feedback for first year medical students. Our goal was to determine whether there are differences in accuracy and learning efficiency between self- regulated and algorithm-regulated groups.

**Methods:**

First year medical students at the University of Calgary completed a dermatologic morphology module. We randomly assigned them to either a self-regulated arm (students removed cases from the practice pool at their discretion) or an algorithm-regulated arm (an algorithm determined when a case would be removed). We then administered a pre-survey, pre-test, post-test, and post-survey. Data collected included mean diagnostic accuracy of the practice sessions and tests, and the time spent practicing. The surveys assessed demographic data and student satisfaction.

**Results:**

Students in the algorithm-regulated arm completed more cases than the self-regulated arm (52.9 vs. 29.3, p<0.001) and spent twice as much time completing the module than the self-regulated participants (34.3 vs. 17.0 min., p<0.001). Mean scores were equivalent between the algorithm- and self-regulated groups for the pre-test (63% vs. 66%, n = 54) and post-test (90% vs. 86%, n = 10), respectively. Both arms demonstrated statistically significant improvement in the post-test.

**Conclusion:**

Both the self-regulated and algorithm-regulated arms improved at post-test. Students spent significantly less time practicing in the self-directed arm, suggesting it was more efficient.

## Introduction

Limited access to dermatology clinic time and a shortage of dermatologists may impede medical students learning of clinical dermatology.^1^ In one study, fewer than 40% of primary care residents felt that their medical school dermatology curriculum properly prepared them to diagnose and treat common skin diseases.^2^ Given the decreasing number of dermatologists practicing in Canada,^3^ adequately preparing medical students to demonstrate competency in dermatology can prove challenging. Clinical teaching of dermatologic pathologies can be inconsistent in medical school. The use of deliberate practice using a library of dermatologic images has the potential to aid in the development of diagnostic skill when clinical settings are not available.

Deliberate practice is defined as repetitive practice accompanied by feedback, gradually increasing in difficulty, and with the goal of obtaining competence or mastery of a skill.^4,5^ A review of deliberate practice in the setting of medical education is well summarized by Ericsson.^6^ When practice is combined with formative feedback, students can learn to monitor, control and evaluate their own performance during independent study.^7^ Several projects have documented the efficacy of deliberate practice in medicine as well as its superiority to conventional clinical education;^8-10^ but research is lacking in dermatology where there is a high degree of visual evidence.^11,12^ Efficiency of learning is the change in performance level (described by serial assessments) divided by the learning investment (time spent and cognitive load).^13^

While learning a skill, the trainee needs to know when they have reached their goals of study or practice. Feedback or assessment is therefore beneficial, and can be provided by a mentor, a pre-defined set of criteria, a quiz or exam, or autonomously determined by the trainee.

We delivered an interactive dermatologic morphology-training module equipped to provide immediate feedback to first-year medical students, thereby meeting the criteria of a deliberate practice activity. The primary goal of this study was to determine whether there were differences in diagnostic accuracy and learning efficiency between a self-regulated versus algorithm-regulated deliberate practice strategy. Our secondary goals were to compare student experiences related to their learning.

## Methods

### Setting and sample size

The University of Calgary Conjoint Health Research Ethics board granted approval for this study. We sent an email describing the study and a secure invitation to join the project to the 165 first year Cumming School of Medicine students, University of Calgary, Canada in December 2016. Students registered a personal profile on www.TeachingMedicine.com and gave consent through an online form.

### Study design and data collection

This was a mixed methods design, consisting of an experimental component and two surveys. Seventy- three students completed an initial demographic survey and a pre-test composed of 13 multiple-choice questions assessing morphology knowledge. One of the authors (DT) created the pre-test by selecting cases that were deemed to represent the range of cases in the curriculum. We excluded these specific cases from the practice library used in the study. We did not provide learners their score or any feedback after the pre-test. Learners were subsequently randomized by computer in double-blind fashion into one of two arms in blocks of four. Students who declined participation in the study project had equal access to the online modules. The medical school did not use data from the modules in their medical school assessment.

### Intervention

The practice module consisted of 63 cases each featuring a photograph of a skin lesion and two questions per case addressing the primary and secondary morphology. To ensure accuracy and internal validity, one dermatologist and one senior dermatology resident reviewed the cases. The review of the cases entailed completing the modules independently and confirming they agreed upon all of the answers to the questions. After the students submitted their response to the practice cases, the program provided them the correct answers immediately.

In the “self-regulated” arm, the student chose to either keep a particular case in the practice pool or remove the case after they submitted their answer and received feedback. In the “algorithm-regulated” arm, the students received a score of +1 for a correct answer and -1 for an incorrect answer (to a minimum score of -1). For example, if the trainee answered twice correctly, they would obtain a score of +2. If they first answered incorrectly (-1), they would need to answer correctly three consecutive times to reach +2. When a case score equalled +2, the case was removed from practice and students were notified. In both study arms, the practice session was complete when all of the cases were removed from the practice pool. We instructed participants to work alone and refrain from utilizing outside reference material during the pre-test, post-test, and practice module. Students were permitted to log in and out of the practice module and their progress would be saved. The website software recorded the time spent viewing the case images and answering the questions. After completion of the practice module and a seven- day lockout period, students completed both a post- test, that was identical to the pre-test, and a learner satisfaction survey. For the post-module survey (see Appendix) questions examining participants’ experience and satisfaction, responses were based on a Likert scale (“Strongly agree” and “Agree” versus “Neutral”, “Disagree” and “Strongly Disagree”).

### Data analysis

We used STATA 10 statistical software for data analysis. Performance data (time and percent correct responses) in the pre-test, practice sessions and post- tests were analyzed using the two sample *t*-test with equal variance. The pre-test and post-test differences were analyzed using paired *t*-tests. The post-test survey data is reported as mean responses expressed as percent of total respondents. Alpha was *a priori* set to 0.05.

## Results

### Participation and demographics

There were no significant differences in the age or gender of participants randomized to the two arms (Table 1). A total of 95.5% of the students had not received any dermatology exposure or training prior to their medical school dermatology block.

**Table 1:** Survey, practice, and test data

Pre-survey Data	Self-Directed	Algorithm-Directed	P value	Both arms
Number of students	35	38		73
Mean Age	27.5 (4.9, 18.4-50.0)	26.9 (4.0, 25.5-28.2)	0.54	
Gender (percent male)	40%	34%	0.61	
Practice is required	35 (100%)	37 (97%)		72 (99%)
Is confident	0 (0%)	2 (5%)		2 (2.8%)
Will be efficient	31 (89%)	31 (82%)		62 (85%)
Will be effective	30 (86%)	30 (79%)		60 (82%)
**Pre-test**				
Number of students	30	24		54
Diagnostic accuracy	65.8 (16.7, 58.7-72.8)	63.3 (16.6, 57.1-69.6)	0.6	
**Practice**				
Number of students	23	29		52
Diagnostic accuracy	70.2 (11.6, 65.0-75.3)	71.8 (8.0, 68.7-74.9)	0.55	
# of cases completed	29.3 (16.3, 16.1-21.8)	52.9 (11.07, 48.6-57.2)	< 0.001	
Minutes practicing	17.0 (11.45, 11.9-22.1)	34.3 (18.0, 27.4-41.4)	< 0.001	
**Post-test**				
Number of students	5	5		10
Diagnostic accuracy	85.8 (7.36, 76.7-94.9)	90 (4.84, 84.0-96.0)	0.32	

Two-point seven percent of participants “agreed” that they felt confident with their skin lesion identification and 98.6% of participants either “agreed” or “strongly agreed” that practice is required for obtaining competency in skin lesion identification.

### Pre-test data

Fifty-four students completed the pre-test (54/165 or 33%). There was no difference in mean accuracy between the self-directed and algorithm-directed arms (65.8% vs. 63.3%, p=0.60, Table 1).

### Practice module data

Fifty students completed the practice module. Students in the algorithm-regulated arm completed more cases than the self-regulated arm (52.9 vs. 29.3, p<0.001, Table 1) and spent twice as much time completing the module than the self-regulated participants (34.3 vs. 17.0 min., p<0.001, Table 1). During practice, we found no significant difference in the accuracy of morphologic identification (71.8% vs. 70.2%, p=0.55, Table 1).

### Post-test data

Only 10 participants completed the post-test, 18.5% of those who completed the pre-test and 6% of all those invited to participate (10/165). All of the study subjects who completed the post-test had also completed the pre-test. There was no statistically significant difference in the mean accuracy between the algorithm vs. the self-regulated groups (90.0 vs. 85.8%, respectively, p= 0.31 and p=0.48) but there was a large effect size (Cohen’s d= 0.8; Table 1).

### Pre-test and post-test comparison

Comparing the pre-test data of the 10 students who also completed the post-test, there was a statistically significant increase in mean diagnostic accuracy on the post-test with a very large effect size (65.4% vs. 87.9, p=0.002, Cohen’s d = 1.91 Table 2).

**Table 2:** Paired post-test vs. pre-test

	Pre-test	Post-test	P value
Number of Students	10	10	
Diagnostic Accuracy	65.4 (15.4, 54.4-76.5)	87.9 (6.3, 83.4-92.4)	0.002

### Post-survey data

The same 10 students who completed the post-test also completed the post-survey. All students (100%) “strongly agreed” or “agreed” that practicing is important for skin lesion identification when compared to textbook or lecture learning. Seventy percent of students agreed that they felt confident with their skin lesion morphology identification skills. All 10 of the students either “strongly agreed” or “agreed” that the instant feedback was important. Eighty percent of the 10 students still felt they needed “a little more practice” and 90% of students “strongly agreed” that they would want to learn other skills using similar practice modules. Seventy percent of students agreed the modules were both effective and fun, and 80% of students agreed that the modules were efficient.

## Discussion

We designed our study to measure differences in diagnostic accuracy and efficiency of learning between an algorithm-regulated arm compared to a self-regulated arm for learning dermatology morphology via deliberate practice. In our study, the algorithm-regulated group spent twice as much time on the practice module and completed 80% more cases than the self-regulated group. However, despite a large effect size, the mean diagnostic accuracy on the post-test was similar for both arms of deliberate practice. While the effect size indicates there may have been a statistically significant difference in scores had our sample size been larger, the marginal degree of improvement in test scores may not warrant the extra amount of time spent practicing. Therefore, the self-directed arm was a more efficient learning strategy. Medical students are faced with increased clinical demands with simultaneously decreased available time to learn or prepare, requiring a focus on efficiency of learning.^14,15^ The change in performance level (described by serial assessments) divided by the learning investment (time spent or cognitive load) is an indication of the efficiency of learning.^13^ Our data suggest that the students in the self-directed arm spent an appropriate amount of time and effort learning dermatology morphology for a short-term retention assessment.

We did observe a statistically significant improvement between the pre-test and post-test mean scores for both groups (65.4% vs. 87.9%, p=0.002, Table 2). Additionally, of the 10 students who completed the post-survey, they all indicated that the practice module improved their confidence in their knowledge of dermatologic morphology. Similar findings of improved confidence amongst medical students were noted in another study implementing a module on the management of common dermatologic conditions.^16^

Students indicated they enjoyed the modules, and found them both effective and efficient. A positive emotional experience of the learner has been shown to increase learner engagement.^17,18^

A study that assessed dermatology knowledge via post-test scores in students who completed online learning with discussion board feedback in addition to didactic teaching found they performed better than students who only completed large group teaching without feedback.^19^ Similar results were noted in two other studies assessing dermatologic knowledge after administration of their online modules.^20,21^ In one study, improved post-test scores were noted following the use of an online curriculum; however, the higher scores correlated with increased use of the curriculum,^22^ which is not what was observed in our study. Of note, two studies reported no significant difference in post-examination scores in students that completed interactive case-based modules vs. the control groups that had not used the interactive design.^23,24^

Other fields of medicine have studied the impact of deliberate practice using computer modules. Ankle x- ray interpretation in the emergency department was studied using over 200 ankle x-rays and trainees were asked to identify the presence or absence of fractures; they describe the learning curve for this skill. Virtual reality simulators using deliberate practice have also been investigated for laryngoscopy and laparoscopy skill acquisition, showing that skills are improved with “virtual” practice.^25,26^

A limitation of our study is that it represents data collected from a single center. Additionally, the power of our study was negatively affected by the small number of students that completed the post- test and post-survey. The effect size of the post-test scores (Cohen’s d=0.8) suggests that despite there being no statistically significant difference, there was an appreciable disparity in scores depending on the intervention. A repeat study with a more robust sample size is necessary to conclusively determine the influence of the two interventions. Furthermore, long-term retention of knowledge was not assessed. Given that the pre- and post-test questions were identical, improved scores may have been secondary to recall bias. Lastly, students who completed the post-test may have been more motivated to learn, which enhanced the effect of the intervention.

### Conclusion

Both the self-regulated and algorithm-regulated deliberate practice strategies were equally effective learning interventions. However, students spent substantially less time and completed fewer cases in the self-directed arm, suggesting the self-directed method was more efficient for this short-term memory task. While our data are not generalizable, it is interesting that the students in the self-regulated arm appear to have adequately assessed when they had completed sufficient practice. Future projects within undergraduate dermatology could incorporate deliberate practice for categorizing lesions as benign or malignant, a common skill that is required for general practitioners, with a focus on optimizing effectiveness, efficiency, learner satisfaction, and additionally, long term retention (a factor that we did not address).

## Appendix

Dermatology Post-Survey

I am confident in my skin lesion identification skills.

- Strongly disagree
- Disagree
- Neutral
- Agree
- Strongly Agree

PRACTICING is required for skin lesion identification:

- Strongly disagree
- Disagree
- Neutral
- Agree
- Strongly Agree

Compared to textbook or lecture learning, PRACTICING is important for skin lesion identification:

- Strongly disagree
- Disagree
- Neutral
- Agree
- Strongly Agree

For my own personal learning needs, how much did I PRACTICE?

- I still need way more practice
- I still need a little more practice
- I practiced about the right amount
- I practiced a little too much
- I practiced way too much

The online PRACTICE modules for skin lesion identification were EFFICIENT (good learning for my time spent)?

- Strongly disagree
- Disagree
- Neutral
- Agree
- Strongly Agree

The online PRACTICE modules for skin lesion identification were EFFECTIVE (they actually worked for me):

- Strongly disagree
- Disagree
- Neutral
- Agree
- Strongly Agree

The instant feedback was important for me:

- Strongly disagree
- Disagree
- Neutral
- Agree
- Strongly Agree

The PRACTICE modules were enjoyable/fun:

- Strongly disagree
- Disagree
- Neutral
- Agree
- Strongly Agree

I would want to learn other skills using online PRACTICE modules:

- Strongly disagree
- Disagree
- Neutral
- Agree
- Strongly Agree

If you have any comments, please tell us:

[free text entry]
